# Promising first‐line immuno‐combination therapies for unresectable hepatocellular carcinoma: A cost‐effectiveness analysis

**DOI:** 10.1002/cam4.70094

**Published:** 2024-08-16

**Authors:** Feng Wen, Peng Huang, Qiuji Wu, Yang Yang, Kexun Zhou, Mengxi Zhang, Qiu Li

**Affiliations:** ^1^ Division of Abdominal Tumor Multimodality Treatment, Department of Radiation Oncology, Cancer Center, West China Hospital Sichuan University Chengdu China; ^2^ Med‐X Center for Informatics Sichuan University Chengdu China; ^3^ Division of Abdominal Tumor Multimodality Treatment, Department of Medical Oncology, Cancer Center, West China Hospital Sichuan University Chengdu China

**Keywords:** cost‐effectiveness, first‐line systemic treatment, immuno‐combination therapy, unresectable hepatocellular carcinoma

## Abstract

**Background and Aims:**

Hepatocellular carcinoma (HCC) is one of the leading causes of cancer‐related death all over the world, and brings a heavy social economic burden especially in China. Several immuno‐combination therapies have shown promising efficacy in the first‐line treatment of unresectable HCC and are widely used in clinical practice. Nevertheless, which combination is the most affordable one is unknown. Our study assessed the cost‐effectiveness of the immuno‐combinations as first‐line treatment for patients with unresectable HCC from the perspective of Chinese payers.

**Methods:**

A Markov model was built according to five multicenter, phase III, open‐label, randomized trials (Himalaya, IMbrave150, ORIENT‐32, CARES‐310, LEAP‐002) to investigate the cost‐effectiveness of tremelimumab plus durvalumab (STRIDE), atezolizumab plus bevacizumab (A + B), sintilimab plus bevacizumab biosimilar (IBI305) (S + B), camrelizumab plus rivoceranib (C + R), and pembrolizumab plus lenvatinib (P + L). Three disease states were included: progression free survival (PFS), progressive disease (PD) as well as death. Medical costs were searched from West China Hospital, published literatures or the Red Book. Cost‐effectiveness ratios (CERs) and incremental cost‐effectiveness ratios (ICERs) were evaluated to compare costs among different combinations. Sensitivity analyses were performed to assess the robust of the model.

**Results:**

The total cost and quality‐adjusted life years (QALYs) of C + R, S + B, P + L, A + B and STRIDE were $12,109.27 and 0.91, $26,961.60 and 1.12, $55,382.53 and 0.83, $70,985.06 and 0.90, $84,589.01 and 0.73, respectively, resulting in the most cost‐effective strategy of C + R with CER of $13,306.89 per QALY followed by S + B with CER of $24,072.86 per QALY. Compared with C + R, the ICER of S + B strategy was $70,725.38 per QALY, which would become the most cost‐effective when the willing‐to‐pay threshold exceeded $73,500/QALY. In the subgroup analysis, with the application of Asia results in Leap‐002 trial, the model results were the same as global data. In the sensitivity analysis, with the variation of parameters, the results were robust.

**Conclusion:**

As one of the promising immuno‐combination therapies in the first‐line systemic treatment of HCC, camrelizumab plus rivoceranib demonstrated the potential to be the most cost‐effective strategy, which warranted further studies to best inform the real‐world clinical practices.

## Graphical abstract

Hepatocellular Carcinoma (HCC) is one of the leading causes of cancer‐related death all over the world, and several immuno‐combination therapies have shown promising efficacy in the first‐line treatment of unresectable HCC. The current study assessed the cost‐effectiveness of the immuno‐combinations as first‐line treatment for patients with unresectable HCC from the Chinese payers' perspective. Our results suggested that camrelizumab plus rivoceranib demonstrated the potential to be the most cost‐effective strategy for patients with no contraindications for immunotherapies, which was a typical representative of immune‐oncology in China.

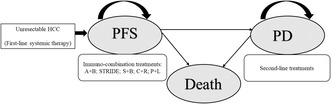

## 1 INTRODUCTION

Hepatocellular carcinoma (HCC) is one of the most commonly cancer‐related health problems all over the world with an increasing incidence and mortality.^1^, ^2^ And about 50%–60% patients are diagnosed at an advanced stage with 5‐year relative survival rate less than 20%.^3^ However, advanced HCC cannot be surgically removed, system therapy is the mainstay of treatment, which could impose a huge economic and health burden globally.^4^

With the emergence of immune checkpoint inhibitors, systemic treatments for advanced HCC have experienced an unprecedented progress over the past several years after the dominant of tyrosine kinase inhibitors(TKIs) for more than a decade.^5^, ^6^ After the groundbreaking results of atezolizumab plus bevacizumab (A + B) in the IMbrave150 trial for unresectable HCC, several immunotherapy based treatments have met their predefined primary endpoints based on multicenter, open‐label, phase III randomized trials, including tremelimumab plus durvalumab (STRIDE) in Himalaya trial, sintilimab plus bevacizumab biosimilar (IBI305) (S + B) in ORIENT‐32 trial, camrelizumab plus rivoceranib (C + R) in CARES‐310 trial.^7^, ^8^, ^9^, ^10^ Therefore, first‐line systemic treatment of advanced HCC has experienced substantial advancement in the past few years especially in China. In addition, the treatment pathway for HCC in China follows global updated guidelines, including National Comprehensive Cancer Network, American Society of Clinical Oncology, American Association for the Study of Liver Diseases, European Society for Medical Oncology as well as Chinese Society of Clinical Oncology, and consistently, all the guidelines have recommended immuno‐combination therapy as the preferred first‐line treatment and TKIs as the alternative option if patients have the contraindications to the immunotherapies.^11^, ^12^, ^13^, ^14^, ^15^ Additionally, though the combination of pembrolizumab plus lenvatinib (P + L) in LEAP‐002 trial did not meet the prespecified dual end points of overall survival (OS) and progression‐free survival (PFS), this strategy is still widely used as first‐line therapy of advanced HCC in China. The reason for choosing this treatment mainly attributed to the increased OS of 21.2 months and objective response rate of 27%.^16^ For Asia patients, the OS reached 26.3 months especially. Besides, the treatment‐related adverse events (AEs) occurred in the lenvatinib plus pembrolizumab group were tolerable.^17^

Due to the lack of the direct comparisons of these approved therapies from the magnitude of clinical benefit, safety and accessibility, it is hard for clinical decision‐makers to provide the recommendations only based on the published guidelines,^18^ and which combination is the most affordable, that would place this treatment in a potential primacy consideration among all the approved immune‐combination treatments is unknown.

As we know, nearly half of the world's HCC burden comes from China. The incidence of HCC ranks fourth with 367,700 new cases in 2022, and HCC‐related death lies second of all cancer‐related death with 316,500 patients died in 2022, bringing tremendous pressure to the health and socio‐economic development.^2^, ^19^ Therefore, it is urgent to provide more comprehensive information about candidate treatment strategies. The current study assessed the cost‐effectiveness of the immuno‐combinations as first‐line treatment for patients with unresectable HCC from the Chinese payers' perspective, the results of which may aid the clinicians in decision‐making in the era of immune‐based regimens.

## 2 MATERIALS AND METHODS

### 2.1 Study design of each trial

For the A + B combination in IMbrave150 trial (NCT03434379), atezolizumab was given at the dose of 1200 mg intravenously every 3 weeks and bevacizumab was 15 mg of per kilogram of body weight intravenously every 3 weeks.^7^ And for STRIDE regimen in Himalaya trial (NCT03298451), tremelimumab was 300 mg for one dose in total and durvalumab was given at the dose of 1500 mg every 4 weeks.^9^ For S + B treatment in ORIENT‐32 trial (NCT03794440), sintilimab was 200 mg every 3 weeks and bevacizumab biosimilar (IBI305) was given at the 15 mg of per kilogram of body weight intravenously every 3 weeks.^8^ For C + R combination in CARES‐310 trial (NCT03764293), camrelizumab was given 200 mg every 2 weeks intravenously and rivoceranib was 250 mg orally once daily.^10^ When it came to P + L strategy in LEAP‐002 trial (NCT03713593), pembrolizumab was given at the dose of 200 mg every 3 weeks and lenvatinib was taken orally 8 mg once a day for patients with body weight less than 60 kg and 12 mg for body weight ≥60 kilograms.^16^ Tumor assessment was conducted at baseline and every 6–9 weeks during the treatment by computed tomography or magnetic resonance imaging until 48–54 weeks, followed by every 9–12 weeks thereafter.

The clinical efficacy, treatment‐related AEs and subsequent regimens were collected from each trial, which was listed in the Table 1. For second‐line treatment, 40.7% patients in STRIDE treatment received it, including 1.8% of immunotherapy, 36.4% of targeted therapy, 1.8% chemotherapy and 0.3% antiangiogenic therapy (bevacizumab). For the A + B group, 18.5% patients had second‐line treatment, including 18.8% of tyrosine kinase inhibitor, 0.6% of angiogenesis inhibitor (monoclonal antibodies), 1.2% of chemotherapy, 1.2% of immunotherapy and 0.6% others. For the S + B group, 29% patients had second‐line treatment, including 22% of systemic therapy other than PD‐(L)1 inhibitors and 2% PD‐(L)1 inhibitors. For the C + R group, 33.1% patients had second‐line treatment, including 27.2% targeted therapy, 1.5% monoclonal antibody, 11.4% immunotherapy and 2.2% chemotherapy. For the P + L group, 44.1% patients had second‐line treatment, including 31.4% targeted therapy, 3.5% monoclonal antibody, 14.4% immunotherapy with/without targeted therapy or monoclonal antibody and 3.5% chemotherapy.

**TABLE 1:** Basic information of five immune‐combination treatments.

	A + B	STRIDE	S + B	C + R	P + L
Simple size	336	393	380	272	395
Median age (range)‐year	64 (56–71)	65 (22–86)	53 (21–82)	58 (48–66)	60 (19–88)
Male sex	82%	83.2%	88%	83%	80.3%
Barcelona Clinic liver cancer stage					
B	15%	19.6%	15%	14%	22%
C	82%	80.4%	85%	86%	78%
Objective response rate	27.3%	20.1%	21%	25.4%	26.1%
Disease control rate	73.6%	60.1%	72%	78.3%	81.3%
PFS time (months)	6.9	3.78	4.6	5.6	8.2
OS time (months)	19.2	16.43	NR	22.1	21.2
Treatment‐related AEs					
Any	98.2%	97.4%	99%	97%	96.5%
Grade 3 or 4	56.8%	50.5%	53%	81%	61.5%
Subsequent treatment	18.5%	40.7%	29%	33.1%	44.1%

Abbreviations: A + B, atezolizumab and bevacizumab combination; AE, adverse event; C + R, camrelizumab and rivoceranib combination; NR, not reached; OS, overall survival; PFS, progression free survival; P + L, pembrolizumab and lenvatinib combination; S + B, sintilimab and bevacizumab biosimilar combination; STRIDE, tremelimumab and durvalumab combination.

### 2.2 Overview of the Markov model

**FIGURE 1:**
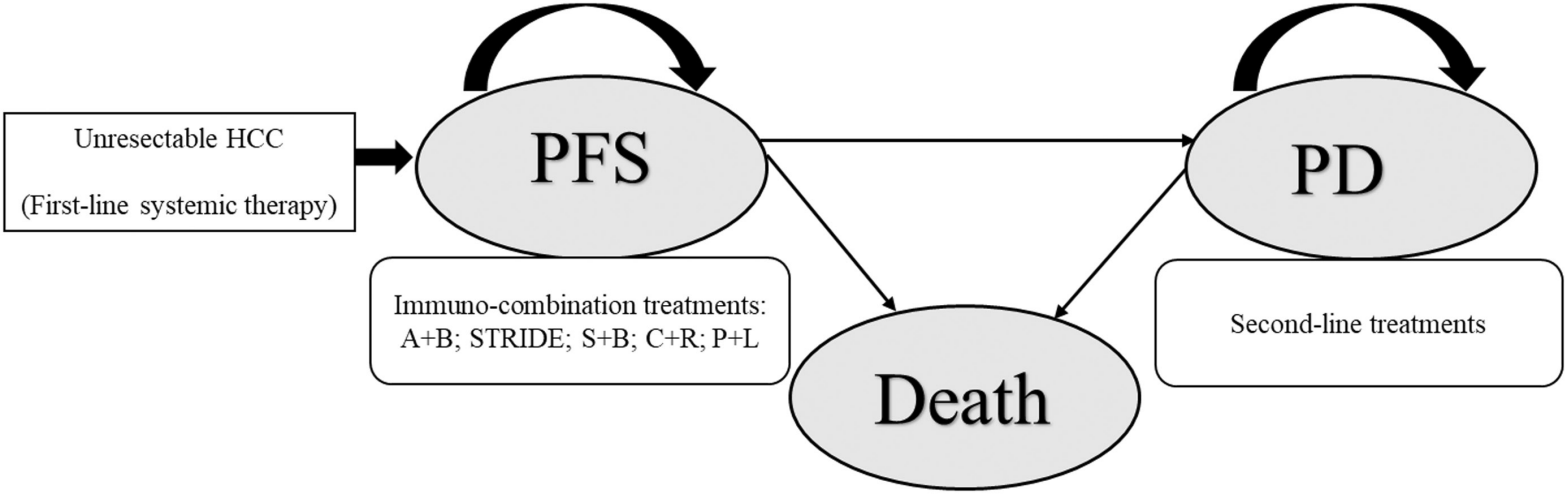
Markov model of five immunotherapy‐based strategies for unresectable HCC. According to the study profile, five groups were analyzed: Group 1, patients with unresectable HCC treated with atezolizumab plus bevacizumab (A + B) combination; Group 2, patients with unresectable HCC treated with tremelimumab plus durvalumab (STRIDE); Group 3, patients with unresectable HCC treated with sintilimab plus bevacizumab biosimilar (IBI305) (S + B); Group 4, patients with unresectable HCC treated with camrelizumab plus rivoceranib (C + R); Group 5, patients with unresectable HCC treated with pembrolizumab plus lenvatinib (P + L). A Markov model comprising three health states (progression free survival, progressive disease and death) was built. HCC, hepatocellular carcinoma; PFS, progression free survival; PD, progression disease.

To comprehensively investigate the therapeutic efficacy and economic consequences among recommended immune‐based combinations in the first‐line treatment of unresectable HCC, a Markov decision model was constructed with the application of Treeage software (Treeage, Williamstown, MA, USA) from the perspective of cost‐effectiveness analysis.^20^, ^21^ Three disease states were included: progression free survival (PFS), progressive disease (PD), and death. All patients were supposed to start in the PFS state and then progress to the PD state or death state according to the transition probabilities (Figure 1). Patients in the PFS state received A + B, STRIDE, S + B, C + R, or P + L as the first‐line treatment until disease progression, intolerable toxicities, or death, then patients would transfer to PD state. In the PD state, partial patients would receive second‐line treatment according to the published information of five clinical trials. If the exact survival numbers of PFS and OS were published, the monthly transition probabilities between each health state were estimated according to the equation: *P* (1 month) = [1˗(0.5)^(1/median time to event)^], which was from *P* = 1−*e*−*R* and *R* = − ln[0.5]/(time to event/number of treatment cycles).^22^ Instead, if the OS value was unknown, such as ORIENT‐32 trial, the transition probabilities were calibrated to best fit the Kaplan–Meier curves of OS and PFS (Figure S1). The model length of transition cycle was 1 month, and the transition probabilities of each combination strategy were listed in Table 2. The time horizon was 10 years, which was the life‐long time for advanced HCC patients.

**TABLE 2:** Transition probabilities, unit costs, and utilities used in the analysis.

	A + B	STRIDE	S + B	C + R	P + L
Probabilities (monthly)					
PFS to PD	0.0656	0.1675	0.0643	0.1164	0.0811
PFS to death	0.0263	0.0414	0.0048	0.0309	0.0322
PD to death	0.0495	0.0534	0.0470	0.0411	0.0519
Costs per cycle ($)					
PFS state					
Atezolizumab	6043.3532	‐	‐	‐	‐
Bevacizumab	2487.3559	‐	‐	‐	‐
Tremelimumab	‐	12,380.9524	‐	‐	‐
Durvalumab	‐	7498.5490	‐	‐	‐
Sintilimab	‐	‐	397.9770	‐	‐
Bevacizumab biosimilar	‐	‐	1850.5928	‐	‐
Rivoceranib	‐	‐	‐	433.9607	‐
Camrelizumab	‐	‐	‐	474.7423	‐
Pembrolizumab	‐	‐	‐	‐	6602.7324
Lenvatinib	‐	‐	‐	‐	1343.1722
Test	175.4512	175.4512	175.4512	175.4512	175.4512
AE	6.7592	12.4781	20.9631	42.0894	21.3019
PD state					
Second‐line therapy	246.9244	937.3418	320.9421	590.1987	125.829
Utility					
PFS state	0.76	0.76	0.76	0.76	0.76
PD state	0.68	0.68	0.68	0.68	0.68
Death state	0.00	0.00	0.00	0.00	0.00

Abbreviations: A + B, atezolizumab and bevacizumab combination; AE, adverse event; C + R, camrelizumab and rivoceranib combination; PD, progressive disease; PFS, progression free survival; P + L, pembrolizumab and lenvatinib combination; S + B, sintilimab and bevacizumab biosimilar combination; STRIDE, tremelimumab and durvalumab combination.

### 2.3 The utilities

Survival time produced in each disease state would be weighted with a utility score to translate into a quality‐adjusted life years (QALYs) related with each treatment, and health‐related quality of life with EQ‐5D usually used to calculate the utility score.^23^ Because of the limited information about EQ‐5D in each trial, the health utility scores for PFS state and PD state were derived from published literatures, and the values were set at 0.76 for PFS state, 0.68 for PD state, and 0 for death state, respectively (Table 2).^20^, ^24^, ^25^

### 2.4 Measurement of costs

Costs were calculated from perspective of Chinese payer, and all the prices were sourced from national price of China or Red book if the drug had no market access in China.^20^, ^26^ Costs for first‐line therapy and second‐line treatment were analyzed as the costs of PFS state and PD state, respectively, and recorded as per patient per month in one transition cycle averagely. For each disease state, treatment‐related direct costs were taken into account, including costs for anti‐tumor drugs, necessary tests costs for efficacy and toxicity during the treatment, and AEs‐related costs. As the second‐line information for five trials was limited, the AEs‐related costs for PD state were ignored. And based on the common use in clinical practice, regorafenib was supposed to be the regimen for all tyrosine kinase inhibitor users in the second‐line treatment (PD state), bevacizumab for angiogenesis inhibitor monoclonal antibodies and oxaliplatin and fluorouracil (FOLFOX) regimen for chemotherapy. An exchange rate of $1 = ￥7.24 (August 3, 2023) was used to convert all costs into US dollars and cost for each item was collected by the time of August 3, 2023, which was relatively stable.

### 2.5 Cost‐effectiveness analysis

The model ran every cycle until all the hypothetical patients moved to death state, and all costs and health outcomes were deducted at a discount of 3% each year.^27^, ^28^ QALYs of given state stands for clinical effectiveness, which was calculated by multiplying the time and utility weight.^29^ Cost‐effectiveness ratios (CERs) indicated the cost per QALY. And incremental cost‐effectiveness ratios (ICERs) implied incremental cost per QALY gained, which was used as the cost‐effectiveness result between two treatments.^28^ The willingness‐to‐pay (WTP) threshold was set at $35,526.90 per QALY (3 × gross domestic product per capita in 2022) for China based on World Health Organization (WHO) guidelines.^30^

### 2.6 Sensitivity analysis

One‐way sensitivity analyses were analyzed to figure out the potential important parameters, and the ranges of the input factors were set at ±20% of the base‐case values.^31^ For cost values, gamma distribution was used and for utility values, beta distribution was used in the analysis. Besides, probabilistic sensitivity analysis was used with stimulating the model for 10,000 iterations with the variations of all the parameters based on the sampling distributions.^28^ And a second‐order monte carlo simulation was modeled to estimate the acceptability probabilities of different optimal strategies with varied WTP thresholds.^32^

## 3 RESULTS

### 3.1 Base‐case results

**FIGURE 2:**
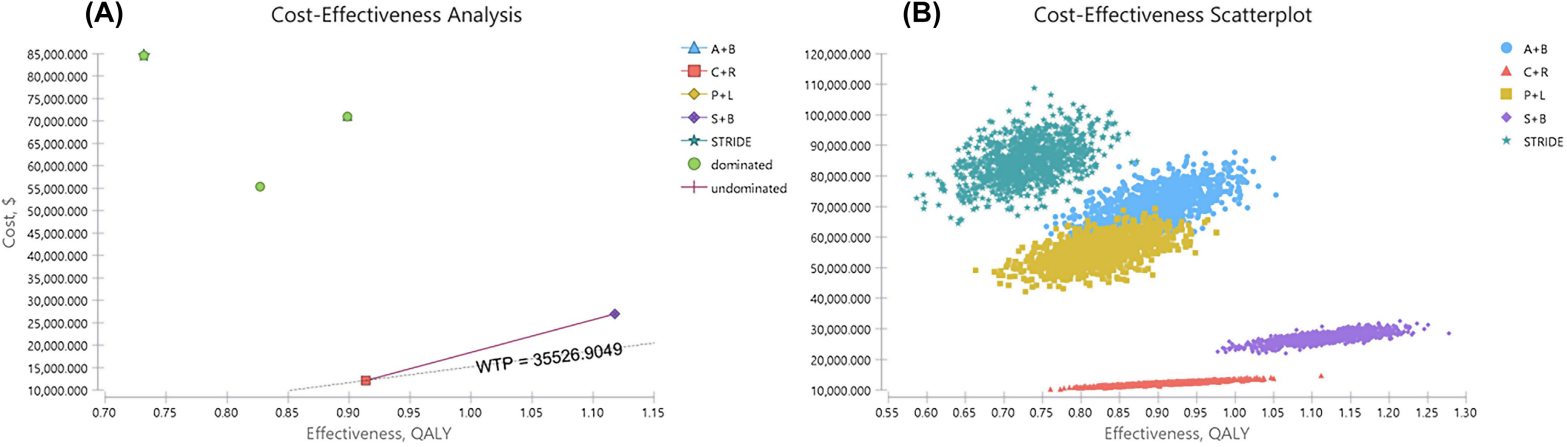
Cost‐effectiveness analysis. (A) The distribution of five strategies. P + L, A + B, and STRIDE were dominated by C + R therapy; (B) The scatterplot of five strategies. A + B, atezolizumab and bevacizumab combination; C + R, camrelizumab and rivoceranib combination; P + L, pembrolizumab and lenvatinib combination; S + B, sintilimab and bevacizumab biosimilar combination; STRIDE, tremelimumab and durvalumab combination; WTP, willingness‐to‐pay.

Based on the model, the total cost and QALYs yielded by treatment of C + R, S + B, P + L, A + B, and STRIDE were $12,109.27 and 0.91, $26,961.60 and 1.12, $55,382.53 and 0.83, $70,985.06 and 0.90, and $84,589.01 and 0.73, respectively, resulting in the most cost‐effective strategy of C + R with CER of $13,306.89 per QALY followed by S + B with CER of $24,072.86 per QALY (Table 3). From the perspective of cost‐effective analysis, P + L, A + B and STRIDE were dominated by C + R therapy (Figure 2A). Compared with C + R, the ICER of S + B strategy was $70,725.38 per QALY, which was unlikely to be a cost‐effective strategy at the current WTP threshold. According to the Monte Carlo simulation, the scatterplot of five strategies were displayed in Figure 2B. In the subgroup analysis, with the application of Asia results in Leap‐002 trial, the clinical efficacy yielded by the P + L was 0.95 QALYs, and the results did not change the preferred strategy, which showed the robust of the models.

**TABLE 3:** Results of the cost‐effectiveness analysis.

	A + B	STRIDE	S + B	C + R	P + L
Costs ($)					
PFS state ($)	69,263.76	76,476.08	24,178.44	6259.67	54,487.29
PD state ($)	1721.30	8112.92	2783.16	5849.59	895.24
Total ($)	70,985.06	84,589.01	26,961.60	12,109.27	55,382.53
Effectiveness (QALYs)					
PFS state	0.50	0.24	0.63	0.35	0.42
PD state	0.40	0.49	0.49	0.56	0.40
Total	0.90	0.73	1.12	0.91	0.83
Cost/effectiveness ratio ($/QALY)	78,872.289	115,887.68	24,072.86	13,306.89	66,725.94

Abbreviations: A‐B, atezolizumab–bevacizumab group; PD, progressive disease; QALYs, quality‐adjusted life years; SD, stable disease; USA, United States of America.

**FIGURE 3:**
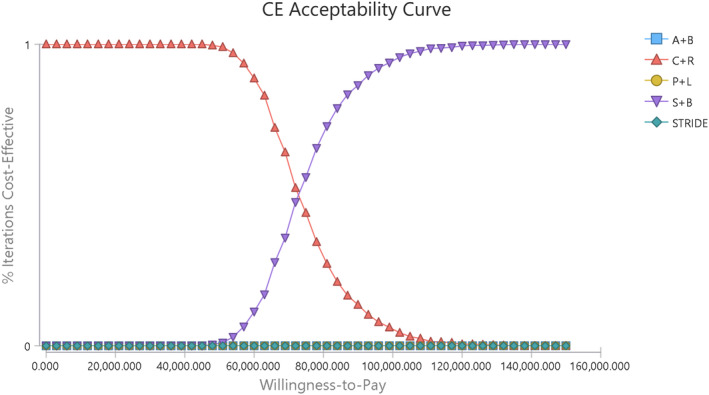
Cost‐effectiveness acceptability curves. The curve indicates with the change of willingness‐to‐pay threshold, the acceptability of five strategies. A + B, atezolizumab and bevacizumab combination; CE, cost‐effectiveness; C + R, camrelizumab and rivoceranib combination; P + L, pembrolizumab and lenvatinib combination; S + B, sintilimab and bevacizumab biosimilar combination; STRIDE, tremelimumab and durvalumab combination.

The cost‐effectiveness acceptability curve revealed with the increase of WTP, the acceptable proportion of C + R kept 100%, then it decreased when the WTP exceeded $39,000/QALY. And the S + B strategy would become the most cost‐effective when the WTP threshold exceeded $73,500/QALY (Figure 3).

### 3.2 Sensitivity analysis

**FIGURE 4:**
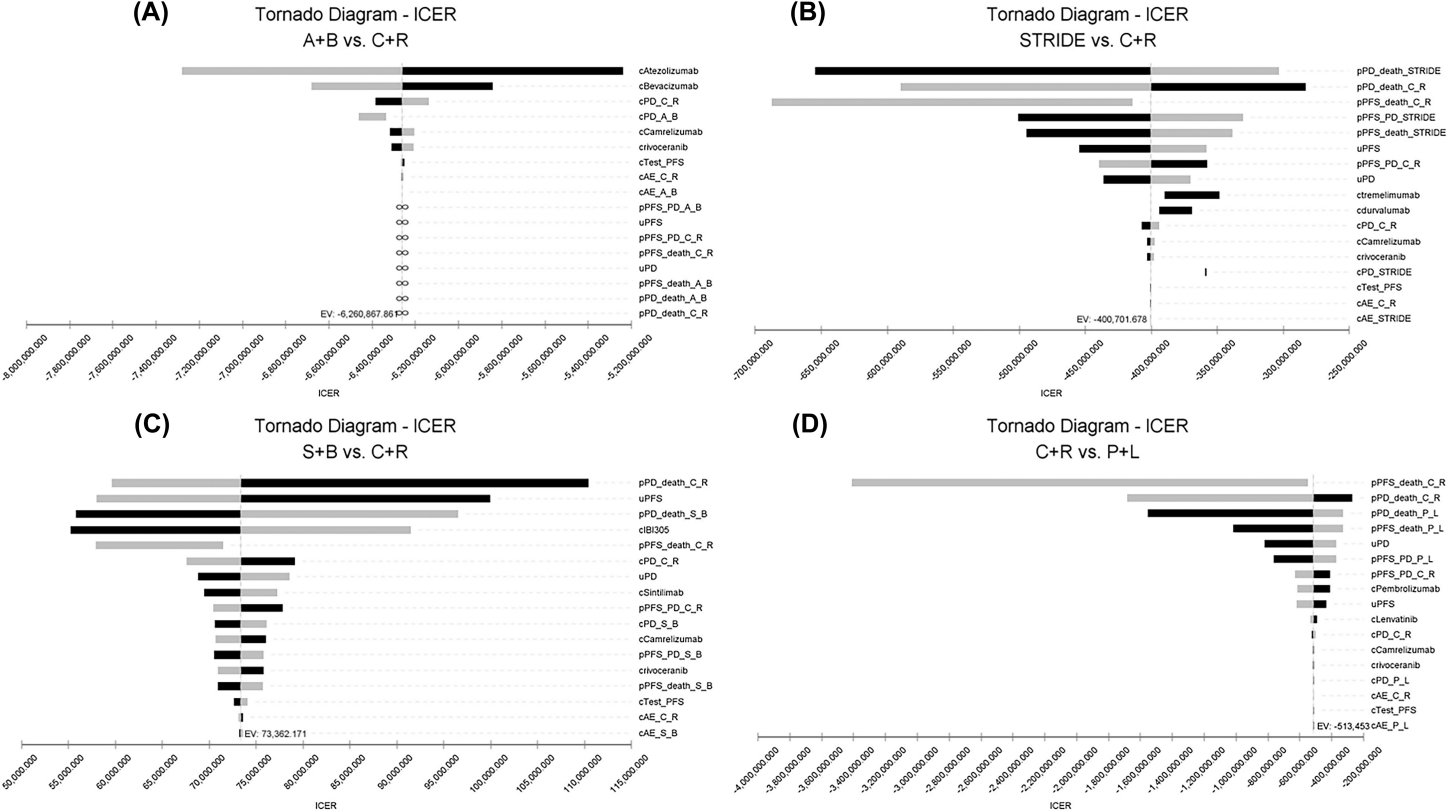
Tornado diagram of sensitivity analysis. The tornado diagrams show the one‐way sensitivity analyses within the variation range for each variable in the comparison of A + B and C + R(A), STRIDE and C + R(B), S + B and C + R(C), as well as P + L and C + R(D), respectively. EV, expected value; ICER, incremental cost‐effectiveness ratios; A + B, atezolizumab and bevacizumab combination; STRIDE, tremelimumab and durvalumab combination; S + B, sintilimab and bevacizumab biosimilar combination; C + R, camrelizumab and rivoceranib combination; P + L, pembrolizumab and lenvatinib combination; uPFS, utility of PFS state; uPD, utility of PD state; PFS, progression‐free survival; PD, progression disease; cAtezolizumab, cost of atezolizumab; cBevacizumab, cost of bevacizumab; cPD_C_R, cost of PD state in the C + R group; pPD_death_STRIDE, transition probability of PD state to death state in the STRIDE group; pPD_death_C_R, transition probability of PD state to death state in the C + R group; pPFS_death_C_R, transition probability of PFS state to death state in the C + R group; pPD_death_S_B, transition probability of PD state to death state in the S + B group; pPD_death_P_L, transition probability of PD state to death state in the P + L group.

As C + R treatment was the dominating choice with lowest CER among five strategies, we set C + R as a reference one. Therefore, one‐way sensitivity analysis was used to compare four other treatments with referenced one to figure out the potential influencing factors of the model and the robustness of the results with the variation of the factors, which were shown as tornado diagram in Figure 4.

In the comparison of A + B and C + R, the cost of atezolizumab was the most influential factors, and with the increase of atezolizumab cost from $4834.68 to $7252.02 per transform cycle, the CER of A + B combination increased from $68,196.30 per QALY to $89,548.27 per QALY, which were still far beyond the threshold of WTP. And the transform probability of PD state to death state in STRIDE regimen (pPD_death_STRIDE) was the most sensitive parameter in STRIDE and C + R comparison. However, the change of pPD_death_STRIDE did not replace the preferred strategy position of C + R. The transform probability of PD state to death state in C + R regimen (pPD_death_C_R) was the most influential factor for S + B and C + R comparison as well as P + L and C + R comparison. And no matter how the parameters changed, the results were robust.

## 4 DISCUSSION

Since the immunotherapy‐based combination brings a new era for the first‐line systemic treatment of unresectable HCC, more and more therapies have gotten their recommendations by guidelines in clinical practice.^33^, ^34^, ^35^ However, whether the combination price reflects its potential benefit from the perspective of cost‐effectiveness remains unknown. In the current study, compared with atezolizumab plus bevacizumab (A + B) in the IMbrave150 trial, tremelimumab plus durvalumab (STRIDE) in Himalaya trial, sintilimab plus bevacizumab biosimilar (IBI305) (S + B) in ORIENT‐32 trial and pembrolizumab plus lenvatinib (P + L) in Leap‐002 trial, camrelizumab plus rivoceranib (C + R) in CARES‐310 trial yielded a total cost of $12,109.27 and efficacy of 0.91 QALYs, resulting in the most cost‐effective strategy with CER of $13,306.89 per QALY followed by S + B with CER of $24,072.86 per QALY.

CARES‐310 trial was a randomized, open‐label, international phase III study conducted in 95 sites over 13 countries and regions all over the world to explore the efficacy and safety of anti‐PD‐1 antibody camrelizumab combined with VEGFR2‐targeted tyrosine‐kinase inhibitor rivoceranib versus sorafenib in the first‐line treatment of HCC. Rivoceranib was given at a low dose of 250 mg orally once daily together with camrelizumab 200 mg intravenously every 2 weeks, and the study results showed a significantly improved PFS (5.6 months vs. 3.7 months; one‐sided *p* < 0·0001) and OS (22.1 months vs. 15.2 months; one‐sided *p* < 0·0001). There were 24% patients suffered from treatment‐related serious AEs.^10^ After the report of successful results, the combination was recommended by CSCO guideline as an option for first‐line treatment of HCC.^15^ However, a recent review with an ad hoc network meta‐analysis indicated that C + R combination was related with a significantly higher risk of treatment‐related AEs compared with A + B combination (relative risk, 1.59; 95% CI, 1.25–2.03; *p* < 0.001).^18^ In the current cost‐effectiveness analysis, we found that the grade 3–4 AEs were 56.8% for A + B, 50.5% for STRIDE, 53.0% for S + B, 81.0% for C + R, and 61.5% for P + L. Correspondingly, the AEs‐related cost per patient per transform cycle was $6.80 for A + B, $12.48 for STRIDE, $20.96 for S + B, $42.09 for C + R, and $21.30 for P + L. However, the lower costs of camrelizumab ($474.74) and rivoceranib ($433.96) per cycle made up for the shortcomings of high cost of AEs in treatment, which may be one of the main reasons leading to C + R combination becoming the dominating strategy.

In the sensitivity analysis, pPD_death_STRIDE (0.0534), pPD_death_C_R (0.0411), and the cost of atezolizumab ($6043.35) were the most influential factors in four comparisons. However, with the variation of these parameters, the results of the model were robust. Because the transform probability was calculated based on the PFS time and OS time, the survival efficacy (pPD_death_STRIDE and pPD_death_C_R) played a crucial role in the results of optimal strategy. Therefore, the C + R group with OS of 22.1 months and PFS of 5.6 months showed advantages over other combinations to some extent. Though Sarah Cappuyns et al. mentioned that A + B combination is considered as the primary standard care in the first‐line settings for advanced HCC,^34^ in our study, the A + B strategy was dominated by C + R group. As in the A + B versus C + R combination, the cost of atezolizumab followed by the cost of bevacizumab were the two most influential factors. With the range of ±20% of the cost of atezolizumab and bevacizumab, the results of the model were not be changed. Even previous study mentioned only 30% discount of bevacizumab price together with 90% discount of primary atezolizumab price, the ICER of A + B compared with sorafenib was the same as the WTP threshold in China.^20^ Hence, A + B combination is still an expensive option as one of the current standard cares for first‐line therapy of HCC, and a price reduction is warranted to make A + B combination more cost‐effective and affordable.

To date, several cost‐effectiveness analyses about the newly approved immunotherapy‐based combinations in the treatment of first‐line HCC have been published. Xu et al. found that from the perspective of Chinese payers, with a WTP threshold of $38,334, sintilimab plus IBI305(S + B) generated an additional $17,552.17 and 0.33 QALYs, resulting in an ICER of $52,817.89 compared to sorafenib, which was unlikely to be cost‐effective for first‐line treatment of HCC.^21^ In summary, most of the cost‐effect analyses for first‐line treatment of HCC indicated neither the A + B nor S + B combination was the cost‐effective option from the perspective of payers in USA and China, which were similar as the results in our study.^21^, ^36^, ^37^, ^38^ To the best of our knowledge, this was the most comprehensive cost‐effectiveness analysis about the promising first‐line immuno‐combination therapies in clinical practice for unresectable hepatocellular carcinoma. As we know, about 50% of the global liver cancer burden were found in China, and more than 70% HCC were hepatitis virus related, in which immunotherapy showed advantage over in non‐hepatitis virus‐related HCC in antitumor efficacy.^2^, ^39^ With these disease characteristics superiority in China, the development of HCC treatment has experienced fast progression. In our model, the drugs in C + R and S + B combinations were made in China which were the two most affordable strategies. Because of the significant deficits of access to essential cancer medicines for HCC, the global appetite for immuno‐oncology regimens has been increasing, especially in low‐ and middle‐income countries. Therefore, even though the C + R and S + B combinations were only approved in China right now, the deliver increasing of these more affordable Chinese immune‐combination drugs may be not far away, which would be an optimal choice for clinicians in decision‐making.^39^

Indeed, there were some drawbacks in our study. Firstly, the five immunotherapy combined strategies were based on the information from multi‐center phase III trials study, which were not a direct cross‐trial comparison. Though the characteristics of inclusion patient were different, but from the perspective of median age, gender ratio and disease stage, there were no big difference. And according to a recent network meta‐analysis, no significant differences were found in overall survival among these treatments.^18^ Hence, a cost‐effectiveness analysis would provide more information for making treatment plan, which warranted further studies to best inform the real‐world clinical practices. Secondly, utilities for the PFS and PD states in our model were collected from published literature and used the same score for five groups, which would affect the results of clinical effectiveness. Usually, utility is calculated based on EQ‐5D results. In addition, compared with other combination, the AEs of C + R treatment was relatively higher, which might result in a lower utility score for C + R treatment. But in the sensitivity analysis, with the variation of AEs‐related cost or utilities for the PFS and PD, the results of the model were robust. Hence, the AEs showed limited effect on the final results of the model. In any case, it is better to collect the EQ‐5D data for each patient to maintain the accuracy of utility scores. Thirdly, the trials of C + R and S + B combination were mainly conducted in China, and the A + B, STRIDE, and P + L were carried out all over the world. The subgroup survival data of China or Asia was better in A + B, STRIDE, and P + L. Because of the limited information, only the subgroup of P + L in Asia was analyzed and the results were consistent with the results in whole population.

In all, this study is the first comprehensive cost‐effectiveness analysis of promising immunotherapy‐based combination therapies in clinical practice as first‐line systemic treatment of unresectable HCC right now. Our results suggest that camrelizumab plus rivoceranib demonstrated the potential to be the most cost‐effective strategy for patients with no contraindications for immunotherapies, which was a typical representative of immune‐oncology in China. Further real‐world studies in different districts are needed to confirm the benefit‐to‐risk of camrelizumab plus rivoceranib combination in clinical practice and steep reductions of other combinations is the direction to make more treatment choice for the first‐line treatment of HCC patients.

## AUTHOR CONTRIBUTIONS

**Feng Wen:** Conceptualization (lead); data curation (lead); formal analysis (lead); funding acquisition (lead); investigation (lead); methodology (lead); project administration (lead); resources (lead); software (lead); supervision (lead); validation (lead); visualization (lead); writing – original draft (lead); writing – review and editing (lead). **Peng Huang:** Data curation (supporting); resources (supporting); software (supporting); validation (supporting). **Qiuji Wu:** Conceptualization (supporting); data curation (supporting); formal analysis (supporting); methodology (supporting); software (supporting). **Yang Yang:** Data curation (supporting); investigation (supporting); methodology (supporting); resources (supporting). **Kexun Zhou:** Investigation (supporting); methodology (supporting); writing – original draft (supporting). **Mengxi Zhang:** Formal analysis (supporting); methodology (supporting); software (supporting); validation (supporting). **Qiu Li:** Conceptualization (lead); formal analysis (lead); methodology (lead); project administration (lead); supervision (lead); validation (lead); visualization (lead); writing – review and editing (lead).

## FUNDING INFORMATION

Science & Technology Department of Sichuan Province, Grant/Award Number: 2022NSFSC1564 and Med‐X Center for Informatics funding project, Sichuan University, Grant/Award Number: YGJC009.

## CONFLICT OF INTEREST STATEMENT

The authors declare no conflict of interest.

## ETHICS STATEMENT

The protocol was approved by the Ethics Committee of West China Hospital, Sichuan University. And a waiver of written informed consent from patients was granted by the Ethics Committee.

## Supporting information


Figure S1.

## Data Availability

The data that support the findings of this study are available from the corresponding author upon reasonable request.
