# Supplementary figures and images for: Promising first‐line immuno‐combination therapies for unresectable hepatocellular carcinoma: A cost‐effectiveness analysis
Source: Cancer Med. 2024 Aug 16;13(16):e70094. doi: 10.1002/cam4.70094 (PMC11327610; doi:10.1002/cam4.70094)

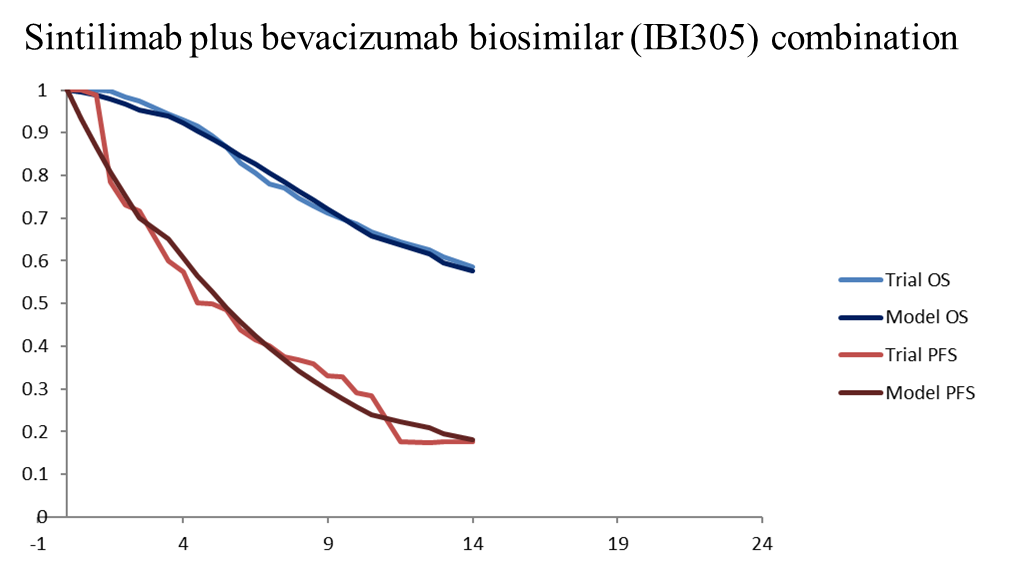

Supplement: Supplementary file 1 — Figure S1. [file CAM4-13-e70094-s001.tif]
